# Household overcrowding and risk of SARS-CoV-2: analysis of the Virus Watch prospective community cohort study in England and Wales

**DOI:** 10.12688/wellcomeopenres.17308.1

**Published:** 2021-12-15

**Authors:** Robert W Aldridge, Helen Pineo, Ellen Fragaszy, Max T Eyre, Jana Kovar, Vincent Nguyen, Sarah Beale, Thomas Byrne, Anna Aryee, Colette Smith, Delan Devakumar, Jonathon Taylor, Srinivasa Vittal Katikireddi, Wing Lam Erica Fong, Cyril Geismar, Parth Patel, Madhumita Shrotri, Isobel Braithwaite, Nicholas Patni, Annalan M.D. Navaratnam, Anne M. Johnson, Andrew Hayward

**Affiliations:** 1 Centre for Public Health Data Science, Institute of Health Informatics, University College London, London, NW1 2DA, UK; 2 Institute for Environmental Design and Engineering, Bartlett School of Environment, Energy and Resources, University College London, London, WC1H 0NN, UK; 3 Institute of Epidemiology and Health Care, University College London, London, UK; 4 Department of Infectious Disease Epidemiology, London School of Hygiene and Tropical Medicine, London, UK; 5 Centre of Health Informatics, Computing and Statistics, Lancaster Medical School, Lancaster University, Lancaster, UK; 6 Liverpool School of Tropical Medicine, Liverpool School of Tropical Medicine, Liverpool, UK; 7 Institute for Global Health, University College London, London, UK; 8 Department of Civil Engineering, Tampere University, Tampere, Finland; 9 MRC/CSO Social and Public Health Sciences Unit, University of Glasgow Institute of Health and Wellbeing,, University of Glasgow, Glasgow, UK; 10 University of Oxford Medical School, Medical Sciences Divisional Office, University of Oxford, Oxford, UK

**Keywords:** COVID-19, Overcrowding, SARS-CoV-2

## Abstract

**Background:** Household overcrowding is associated with increased risk of infectious diseases across contexts and countries. Limited data exist linking household overcrowding and risk of COVID-19. We used data collected from the Virus Watch cohort to examine the association between overcrowded households and SARS-CoV-2.

**Methods:** The Virus Watch study is a household community cohort of acute respiratory infections in England and Wales. We calculated overcrowding using the measure of persons per room for each household. We considered two primary outcomes: PCR-confirmed positive SARS-CoV-2 antigen tests and laboratory-confirmed SARS-CoV-2 antibodies. We used mixed-effects logistic regression models that accounted for household structure to estimate the association between household overcrowding and SARS-CoV-2 infection.

**Results:**26,367 participants were included in our analyses. The proportion of participants with a positive SARS-CoV-2 PCR result was highest in the overcrowded group (9.0%; 99/1,100) and lowest in the under-occupied group (4.2%; 980/23,196). In a mixed-effects logistic regression model, we found strong evidence of an increased odds of a positive PCR SARS-CoV-2 antigen result (odds ratio 2.45; 95% CI:1.43–4.19; p-value=0.001) and increased odds of a positive SARS-CoV-2 antibody result in individuals living in overcrowded houses (3.32; 95% CI:1.54–7.15; p-value<0.001) compared with people living in under-occupied houses.

**Conclusion:**Public health interventions to prevent and stop the spread of SARS-CoV-2 should consider the risk of infection for people living in overcrowded households and pay greater attention to reducing household transmission.

## Introduction

Household overcrowding is associated with increased risk of infectious diseases across cultures and countries ^1^. The World Health Organization Housing and Health Guidelines emphasise the health risks of overcrowding and note its complex economic, social and political determinants ^2, 3^. Several definitions of overcrowding exist and it is also used as an indicator of material deprivation ^4^. Measures of overcrowding assess whether there is adequate dwelling space for occupants’ needs related to shelter, space and privacy ^1^.

According to the English Housing Survey, approximately 787,000 (3%) of English households are overcrowded with unequal distribution across regions and social groups ^5^. A total of 7% of the most deprived households were overcrowded compared with less than half a per cent of the least deprived households. Overcrowding was highest in London compared with all other English regions. White British households are less likely to be overcrowded than households from all other ethnic groups.

There is strong evidence that household overcrowding is associated with risk of infectious diseases, such as tuberculosis ^1^, and increasing evidence on the association between household overcrowding and coronavirus disease 2019 (COVID-19). Studies from Europe have not found a consistent association: Swedish registry data ^6^ revealed increased COVID-19 mortality among over 70 year olds from households, or care homes, with fewer square metres per inhabitant, but a retrospective cohort in Spain did not. In the USA, an ecological analysis found that COVID-19 death rates were higher in the counties with highest percentage of household crowding (16.8 per 100,000) compared with the least crowded areas (4.9 per 100,000) ^7^. A study using 2011 UK census data examined ethnicity, household composition and COVID-19 mortality and found that elderly adults living with younger people were at increased risk of COVID-19 mortality ^8^. The study described the distribution of overcrowded housing within census data used, but it did not adjust for overcrowded housing in the causal mediation analysis as these were considered consequences of living in a multi-generational household rather than confounding factors. A study of UK Biobank data found household size was associated with COVID-19 infection after adjusting for age, sex, deprivation, ethnicity and body mass index (BMI) ^9^. The REACT study found severe acute respiratory syndrome coronavirus 2 (SARS‑CoV‑2) antibodies increased from 4.7% for people in single-person households to 13% in households of seven or more ^10^. The OpenSAFELY study found household size accounted for 10–16% of the excess risk of testing positive for SARS-CoV-2 and 12–39% of the excess risk of COVID-19 mortality in South Asian groups ^11^.

Relatively few studies have investigated the impact of overcrowding specifically, as opposed to household size. In Birmingham (UK), an analysis of data from 408 hospitalised COVID-19 patients found that people from areas of the city with low housing quality and overcrowding were more likely to be admitted to intensive care compared with other areas ^12^. A study of ≥70 year olds using Swedish cause of death, administrative and dwelling registry data corroborates this finding with individual-level overcrowding data. After adjusting for individual age, sex, country of birth, income and education, COVID-19 mortality was 2.1-times (HR 2.1, 95% CI 1.53–2.87) higher among ≥70 year olds in households with less than 20 m ^2^/inhabitant compared with those with >60 m ^2^/inhabitant ^6^. In a serological survey undertaken in Lima, Peru, lateral-flow SARS-CoV-2 seropositivity was also more prevalent among households in the two most overcrowded quartiles compared with the least overcrowded quartile (respective prevalence ratios 1.41 and 1.99, 95% CI 1.01–1.97 and 1.41–2.81) as measured by a ratio of inhabitants to habitable rooms with adjustment for sex, age group, local region and socio-economic status ^13^.

Overcrowding, as defined by more than three household members with fewer than six rooms between them, carried increased odds of laboratory-confirmed secondary infection of SARS-CoV-2 in 92 North Carolinian households within 28 days of a PCR-confirmed index case. Higher rates of household overcrowding observed among non-White/Hispanic people were also argued to account for the higher rates of secondary transmission found among those ethnic groups in this study ^14^.

Additional evidence on the association between household overcrowding and COVID-19 risk can guide immediate public health mitigation measures to reduce the spread of the SARS-CoV-2 and inform long-term housing policy in the UK. In this analysis we aim to examine the association between overcrowded households and either polymerase chain reaction (PCR) confirmed SARS-CoV-2 or antibodies acquired through SARS-CoV-2 infection ^15^.

## Methods

The Virus Watch study is a household community cohort of acute respiratory infections in England and Wales that started recruitment in June 2020 ^15^. As of 28th February 2021, there were 46,937 participants in Virus Watch. A detailed description of recruitment methods has been described previously, but in summary, to recruit our sample we used a range of methods. We used the Royal Mail Post Office Address File to generate a random list of residential address lists that were sent recruitment postcards (n=3,914), we placed social media adverts on Facebook and Twitter (n=18,594), sent SMS messages (n=11,151) and letters to participants from their General Practitioners (n=3,803). Participants were followed up weekly by email with a link to an illness survey which asks about the presence or absence of symptoms that could indicate COVID-19 disease including respiratory, gastrointestinal and general infection symptoms. The weekly survey is also used to capture SARS-CoV-2 test results received from outside the study ( *e.g. via* the UK test, trace and isolate system).

### Laboratory cohort

Nested within this larger study is a sub-cohort of 10,330 adults (aged over 18 years) participating in monthly antibody testing who completed at-home capillary blood sampling kits sent *via* post on a monthly basis, and provided self-reported vaccination data on a weekly basis, in addition to demographic and clinical data at baseline. Individuals were included in this analysis if they underwent antibody testing between 1st February 2021 and 28th August 2021 and completed the February 2021 monthly survey.

### Monthly survey

The Virus Watch monthly survey includes demographic, psychosocial/behavioral, environmental and health-related questions beyond the scope of the weekly survey. Data used in the analysis in this manuscript are taken from the Virus Watch third monthly survey that was sent to participants on 9th February 2021 and occurred during the third national lockdown for both England and Wales. In this survey, we included a series of questions about participants’ housing status adapted from housing-related items in the 2011 England and Wales Census as this the most comprehensive study in terms of coverage of the housing characteristics collected. The survey items collected comprised of: accommodation type; whether accommodation was self-contained; number of rooms available for exclusive use by the household, excluding bathrooms, toilets, halls or landings, or rooms that can be used only for storage ( *e.g*. cupboards); number of bedrooms (built or converted for use as bedrooms, even if not currently used as a bedroom); housing tenure and, if rented, details of the rental arrangement; and whether the accommodation had central heating. Additionally, we presented binary (yes/no) questions about whether participants’ accommodation had visible mould or fungus and/or damp spots on the walls or ceiling due to their association with respiratory tract infections ^16, 17^. Housing-related survey questions are presented in full in the supplementary appendix in the extended data.

### Overcrowding measure

To investigate overcrowding, we first calculated the persons per room ^4^ for each household, which is defined as the number of household occupants divided by the number of rooms, excluding kitchen or bathrooms. We chose persons per room for several reasons, including: it aligned with the data we collected in our monthly survey which was based upon the 2011 UK Census question items; and it is a widely used and valid measure of overcrowding ^18^; and it has been found to have fair agreement with other measures of overcrowding ^4^. We did not ask participants to exclude kitchens from their reporting of the number of rooms in their accommodation and therefore we have assumed that all accommodation had a kitchen and subtracted one from the total number of rooms reported by each household. As described in previous studies examining the validity persons per room as a measure of overcrowding ^4^, we categorised it as: 1) under-occupied accommodation where the number of rooms was greater than the number of  people; 2) balanced accommodation where the number of rooms was equal to the number of people; 3) overcrowded accommodation where the number of rooms was fewer than the number of people. 

### Outcomes

We considered two primary outcomes. First, we were able to determine PCR-confirmed SARS-CoV-2 disease in all participants tested by the national surveillance system in community settings from March 2020 to August 2021. Positive SARS-CoV-2 results were identified from linkage of patient demographic characteristics (name, date of birth, address, NHS number) to the national Second Generation Surveillance System for SARS-CoV-2. Second, we examined laboratory-confirmed SARS-CoV-2 antibodies acquired through infection, among a subset of participants who underwent antibody testing between February 2021 to August 2021. The main outcome variable was evidence of prior infection defined as a cut off index of 0.1 or more on the Roche Elecsys anti-N total immunoglobulin assay that measures seropositive for the Nucleocapsid protein.

### Covariates

Our analysis strategy was informed by conceptual models (from BMJ and the UK government) that have previously described the possible pathways between ethnicity and socio-economic status, overcrowding and risk of SARS-CoV-2 infection and we developed a directed acyclic graph to inform covariate selection (Supplementary Appendix Figure S1 in the extended data). We used this and the rules outlined by VanderWeele as principles of confounder selection ^19^. In our primary analysis, we considered age, sex, ethnicity, household income and geographical region as covariates (Model A). Age, sex, ethnicity and geographical region were derived from participants’ responses to demographic questions at study baseline. Household income was derived from the February 2021 monthly survey.

### Statistical analyses

We undertook description of the characteristics of included participants. To model the association between the selected covariates and each outcome, we conducted univariable and multivariable analyses using mixed-effects logistic regression models with a household-level random effect to account for household-level variation not explained by the covariates using the glmer function in R 4.0.3 (RRID:SCR_001905).

### Sensitivity analyses

We have examined our assumption regarding kitchens in our calculation of overcrowding, by only excluding one room from households with two or more rooms in a sensitivity analysis. In addition to the primary mixed-effects model, we ran three other separate sensitivity analyses (all accounting for household-level clustering): first, a minimal sufficient adjustment set informed by our directed acyclic graph (Model B); second, a model with age, sex, households with children (a binary variable representing households with and without children), and number of close contacts outside the household as covariates (repeating an analysis previously reported prior to having occupational category or household income data available (Model C); third, a model with age, sex, ethnicity, income and occupation as covariates (Model D).

### Ethics

The Virus Watch study has been approved by the Hampstead NHS Health Research Authority Ethics Committee. Ethics approval number - 20/HRA/2320. Written informed consent for the collection and use of data was obtained from participants.

### Role of the funding source

The study sponsor(s) had no role in study design; in the collection, analysis, and interpretation of data; in the writing of the report; and in the decision to submit the paper for publication. We confirm that all authors accept responsibility to submit for publication. For information security reasons, RWA, EF, ME, JC, VN, SB, TB, AA, WLEF, CG, PP, MS, AMDN and AH had full access to all individual level data in the study analyses and all other authors had access to aggregated data.

## Results

On 9th February 2021, participants in the Virus Watch study were invited to take part in the monthly survey. We include responses to the survey for 26,367 participants (56.2%; 26,367/46,937) in our primary analyses. The median number of rooms per household was 6 (interquartile range 5–7) and the median number of householders was 2 (interquartile range 1–3). 4.2% of participants (1,100/26,367) were classified as living in overcrowded households and 7.9% (2,071/26,367) in balanced households. Participants responding to the monthly survey containing housing related questions were more likely to be over 65, of White British ethnicity and live alone or with one other than the general population ( Table 1).

**Table 1.** Description of Virus Watch participants on 28th February 2021, comparing all Virus Watch participants to those who took part in the February 2021 survey and ONS census data for England and Wales.

Characteristic	All Virus Watch participants on 28th Feb 2021	Virus Watch Participants completing monthly survey in Feb 2021	ONS * (%)
All	46,937 (100%)	26,364 (100%)	(100%)
Age group			
0–15	5,866 (12%)	2,356 (8.9%)	(19.1%)
16–24	2,698 (5.7%)	1,104 (4.2%)	(10.6%)
25–44	9,123 (19%)	3,511 (13%)	(26.1%)
45–64	15,781 (34%)	9,365 (36%)	(25.6%)
65+	13,469 (29%)	10,028 (38%)	(18.5%)
Ethnicity			
White British	33,533 (71%)	22,712 (86%)	(80.5%)
White Irish	585 (1.2%)	370 (1.4%)	(0.9%)
White Other	2,117 (4.5%)	1,245 (4.7%)	(4.4%)
Mixed	776 (1.7%)	422 (1.6%)	(2.2%)
South Asian	1,576 (3.4%)	597 (2.3%)	(5.3%)
Other Asian	368 (0.8%)	191 (0.7%)	(2.2%)
Black	256 (0.5%)	113 (0.4%)	(3.3%)
Other Ethnicity	201 (0.4%)	106 (0.4%)	(1%)
Prefer not to say	116 (0.2%)	47 (0.2%)	-
Missing	7,409 (16%)	561 (2.1%)	-
Sex **			
Male	17,652 (38%)	11,733 (45%)	(49.4%)
Female	21,982 (47%)	14,138 (54%)	(50.6%)
Missing/Suppressed	7303 (16%)	493 (1.9%)	-
Number of householders ***			
1	7,174 (15%)	4,672 (18%)	(29.5%)
2	20,821 (44%)	13,802 (52%)	(34.5%)
3	7,047 (15%)	3,363 (13%)	(15.4%)
4	8,146 (17%)	3,345 (13%)	(13.9%)
5	2,783 (5.9%)	960 (3.6%)	(4.5%)
6	966 (2.1%)	222 (0.8%)	(2.1%)
Region			
East Midlands	3,690 (7.9%)	2,352 (8.9%)	(4.5%)
East of England	9,308 (20%)	5,799 (22%)	(12.4%)
London	6,732 (14%)	3,390 (13%)	(9.3%)
North East	2,156 (4.6%)	1,278 (4.8%)	(8.1%)
North West	4,614 (9.8%)	2,809 (11%)	(10%)
South East	8,096 (17%)	5,049 (19%)	(10.5%)
South West	3,011 (6.4%)	1,945 (7.4%)	(15.1%)
Wales	1,042 (2.2%)	593 (2.2%)	(15.4%)
West Midlands	2,318 (4.9%)	1,434 (5.4%)	(9.5%)
Yorkshire and The Humber	2,056 (4.4%)	1,275 (4.8%)	(5.3%)
Missing	3,914 (8.3%)	440 (1.7%)	-
Household overcrowding category ****			
Under occupied	23,196 (88%)	23,195 (88%)	
Balanced	2,071 (7.9%)	2,070 (7.9%)	
Overcrowded	1,100 (4.2%)	1,099 (4.2%)	(3%)
Missing	20,570	-	

*ONS data for age and region drawn from Mid-2019 Estimates of the Population for the UK, England, and Wales, Scotland and Northern Ireland (figures for England and Wales). ** We suppressed some groups due to deductive disclosure in some groups ***ONS data for household size drawn from Families and Households in the UK 2019 (UK wide estimates). **** ONS overcrowded households figures from https://www.ethnicity-facts-figures.service.gov.uk/housing/housing-conditions/overcrowded-households/latest Note: the total number of participants in Table 1 and Table 2 differs because a small number of participants completed the Feb 2021 survey after March 2021

Between 8th March 2020 and 10th August 2021, 2,689 Virus Watch participants (5.7%; 2,689/46,937) had a positive SARS-CoV-2 antigen test through the national test and trace system. 1,229 participants with a PCR confirmed SARS-CoV-2 antigen test were included (4.9%; 1,229/26,367). The proportion of participants with a positive SARS-CoV-2 antigen result was highest in the overcrowded group (9.0%; 99/1,100) and lowest in the under-occupied group (4.2%; 980/23,196); Table 2).

**Table 2.** Description of SARS-CoV2 PCR (up to 10th August 2021) and antibody test results (up to 28th August 2021) for housing and demographic characteristics of Virus Watch participants.

	SARS-CoV2 PCR		SARS-CoV2 antibody	
	Negative / Not tested	Positive	Negative	Positive
All	25,138 (95%)	1,229 (4.9%)	9,672 (91%)	990 (9.3%)
Household overcrowding category				
Under occupied	22,216 (96%)	980 (4.2%)	8,995 (92%)	830 (8.4%)
Balanced	1,921 (93%)	150 (7.2%)	495 (82%)	105 (18%)
Overcrowded	1,001 (91%)	99 (9.0%)	182 (77%)	55 (23%)
Age Group				
0–15	2,209 (94%)	147 (6.2%)	-	-
16–24	983 (89%)	121 (11%)	167 (81%)	39 (19%)
25–44	3,243 (92%)	269 (7.7%)	1,052 (84%)	202 (16%)
45–64	8,898 (95%)	468 (5.0%)	3,835 (89%)	462 (11%)
65+	9,805 (98%)	224 (2.2%)	4,618 (94%)	287 (5.9%)
Sex				
Male	11,220 (96%)	514 (4.4%)	4,151 (91%)	421 (9.2%)
Female	13,440 (95%)	700 (5.0%)	5,506 (91%)	566 (9.3%)
Missing/suppressed	478 (97%)	15 (3.0%)	15(83%)	3 (17%)
Ethnicity				
White British	21,666 (95%)	1,049 (4.6%)	8,859 (91%)	843 (8.7%)
White Irish	357 (96%)	13 (3.5%)	127 (89%)	16 (11%)
White Other	1,190 (96%)	55 (4.4%)	386 (86%)	63 (14%)
Mixed	391 (93%)	31 (7.3%)	71 (79%)	19 (21%)
South Asian	552 (92%)	45 (7.5%)	104 (75%)	34 (25%)
Other Asian	182 (95%)	9 (4.7%)	57 (92%)	5 (8.1%)
Black	107 (95%)	6 (5.3%)	26 (84%)	5 (16%)
Other Ethnicity	101 (95%)	5 (4.7%)	31 (91%)	3 (8.8%)
Prefer not to say	45 (96%)	2 (4.3%)	11 (85%)	2 (15%)
Missing	547 (98%)	14 (2.5%)	0 (NA%)	0 (NA%)
Household income combined for last 12 months				
£0-9,999	847 (95%)	44 (4.9%)	321 (90%)	36 (10%)
£10,000-24,999	4,526 (96%)	206 (4.4%)	2,059 (93%)	154 (7.0%)
£25,000-49,999	7,275 (96%)	327 (4.3%)	3,206 (91%)	304 (8.7%)
£50,000-£74,999	3,970 (95%)	227 (5.4%)	1,515 (88%)	205 (12%)
£75,000-£99,999	2,017 (95%)	106 (5.0%)	676 (88%)	90 (12%)
£100,000+	2,103 (95%)	122 (5.5%)	666 (88%)	88 (12%)
Prefer not to say	1,336 (96%)	52 (3.7%)	571 (93%)	41 (6.7%)
Missing	3,064 (95%)	145 (4.5%)	658 (90%)	72 (9.9%)
Geographical region				
East Midlands	2,230 (95%)	122 (5.2%)	865 (92%)	77 (8.2%)
East of England	5,548 (96%)	252 (4.3%)	2,302 (92%)	193 (7.7%)
London	3,183 (94%)	207 (6.1%)	963 (81%)	232 (19%)
North East	1,209 (95%)	69 (5.4%)	494 (93%)	38 (7.1%)
North West	2,646 (94%)	164 (5.8%)	1,031 (89%)	129 (11%)
South East	4,843 (96%)	206 (4.1%)	1,998 (93%)	153 (7.1%)
South West	1,877 (97%)	68 (3.5%)	758 (95%)	38 (4.8%)
Wales	593 (100%)	0 (0%)	209 (93%)	16 (7.1%)
West Midlands	1,378 (96%)	57 (4.0%)	539 (91%)	54 (9.1%)
Yorkshire and The Humber	1,194 (94%)	81 (6.4%)	506 (90%)	59 (10%)
Missing	437(99%)	3(0.7%)	7(88%)	1(12%)

Note: the total number of participants in Table 1 and Table 2 differs because a small number of participants completed the Feb 2021 survey after March 2021.

In a mixed-effects logistic regression model that included age, sex, ethnicity, household income and geographical region as fixed effects and a household-level random effect we found strong evidence of an increased odds of having a positive SARS-CoV-2 antigen result in individuals living in overcrowded accommodation (odds ratio (OR): 2.45; 95% CI:1.43–4.19; p-value=0.001) and those in balanced homes (OR: 1.72; 95% CI: 1.13–2.63; p-value=0.012) compared with those in under-occupied homes ( Table 3). The proportion of variation at the household level ( *i.e.* intracluster correlation coefficient, or ICC) was 64.9%.

**Table 3.** Univariable and multivariable mixed effects logistic regression models examining the association between household overcrowding and SARS-CoV-2 PCR test results.

Characteristic	Univariable logistic regression			Multivariable logistic regression		
	OR	95% CI	p-value	OR	95% CI	p-value
Household overcrowding category						
Under occupied	1.00	— —	—	1.00	— —	—
Balanced	2.48	(1.76, 3.49)	<0.001	1.72	(1.13, 2.63)	0.012
Overcrowded	3.89	(2.47, 6.13	<0.001	2.45	(1.43, 4.19)	0.001
Age Group						
0–15	3.87	(2.82, 5.30)	<0.001	3.55	(2.31, 5.45)	<0.001
16–24	9.31	(6.54. 13.25)	<0.001	9.17	(5.87, 14.32)	<0.001
25–44	6.13	(4.63, 8.11)	<0.001	5.65	(3.78, 8.44)	<0.001
45–64	3.07	(2.44, 3.87)	<0.001	3.04	(2.13, 4.34)	<0.001
65+	1.00	— —	—	1.00	— —	—
Sex						
Male	1.00	— —	—	1.00	— —	—
Female	1.19	(1.03, 1.38)	0.02	1.17	(0.97, 1.42)	0.10
Missing	1.14	(0.45, 2.90)	0.78	5.66	(0.10, 335.64)	0.41
Ethnicity						
White British	1.00	— —	—	1.00	— —	—
White Irish	0.82	(0.39, 1.72)	0.60	0.78	(0.28, 2.14)	0.63
White Other	0.86	(0.56, 1.31)	0.48	0.44	(0.24, 0.80)	0.007
Mixed	1.51	(0.85, 2.69)	0.16	0.86	(0.44, 1.68)	0.66
South Asian	2.05	(1.16, 3.62)	0.013	1.00	(0.49, 2.04)	0.99
Other Asian	0.89	(0.33, 2.44)	0.83	0.50	(0.14, 1.80)	0.29
Black	1.25	(0.37, 4.30)	0.72	0.66	(0.14, 3.10)	0.60
Other Ethnicity	1.12	(0.32, 3.87)	0.859	0.58	(0.12, 2.81)	0.50
Missing	0.71	(0.31, 1.62)	0.418	0.13	(0.00, 6.53)	0.305
Household income combined for last 12 months						
£0-9,999	1.28	(0.76, 2.16)	0.36	0.98	(0.47, 2.06)	0.964
£10,000-24,999	1.00	— —	—	1.00	— —	—
£25,000-49,999	0.94	(0.71, 1.25)	0.668	0.81	(0.54, 1.23)	0.332
£50,000-£74,999	1.38	(1.00, 1.91)	0.05	0.91	(0.58, 1.43)	0.68
£75,000-£99,999	1.26	(0.84, 1.90)	0.27	0.73	(0.42, 1.29)	0.28
£100,000+	1.60	(1.07, 2.40)	0.02	0.90	(0.52, 1.54)	0.693
Prefer not to say	0.74	(0.45, 1.23)	0.249	0.59	(0.28, 1.25)	0.168
Missing	1.20	(0.84, 1.72)	0.315	0.99	(0.59, 1.64)	0.957
Geographical region						
East of England	1.00	— —	—	1.00	— —	—
East Midlands	1.31	(0.90, 1.91)	0.15	1.32	(0.78, 2.22)	0.30
London	1.89	(1.37, 2.62)	<0.001	1.48	(0.93, 2.37)	0.098
North East	1.49	(0.94, 2.37)	0.09	1.51	(0.81, 2.83)	0.20
North West	1.66	(1.17, 2.34)	0.004	1.68	(1.05, 2.69)	0.032
South East	0.97	(0.71, 1.32)	0.845	0.95	(0.61, 1.48)	0.820
South West	0.79	(0.51, 1.21)	0.27	0.81	(0.43, 1.55)	0.53
West Midlands	1.05	(0.66, 1.66)	0.85	1.08	(0.55, 2.09)	0.83
Yorkshire and The Humber	1.91	(1.22, 2.98)	0.005	2.04	(1.13, 3.70)	0.019

Between 26th February 2021 and 28th August 2021, we had SARS-CoV-2 anti-N total immunoglobulin assay antibody results for 10,662 (23%; 10,662/46,937) participants with housing data ( Table 2). The proportion of participants with a positive SARS-CoV-2 antibody result was highest in the overcrowded group (23%; 55/237) and lowest in the under-occupied group (8.4%; 830/9,825; Table 2). In a mixed-effects logistic regression model that included age, sex, ethnicity, household income and geographical region as fixed effects and a household-level random effect, we found evidence of an increased odds of having a positive SARS-CoV-2 antibody result in individuals living in overcrowded houses (OR: 3.32; 95% CI: 1.54–7.15; p-value<0.001) and in those living in balanced houses (OR: 2.64; 95% CI: 1.57–4.43; p-value<0.001) compared with people living in under-occupied houses ( Table 4). The proportion of variation at the household level (ICC) was 74.0%.

**Table 4.** Univariable and multivariable mixed effects logistic regression models examining the association between household overcrowding and SARS-CoV-2 antibody test results.

Characteristic	Univariable logistic regression			Multivariable logistic regression		
	OR	95% CI	p-value	OR	95% CI	p-value
Household overcrowding category						
Under occupied	1.00	— —	—	1.00	— —	—
Balanced	5.01	(3.13, 8.01)	<0.001	2.64	(1.57, 4.43)	<0.001
Overcrowded	8.20	(4.04, 16.66)	<0.001	3.32	(1.54, 7.15)	<0.001
Age group						
18–24	9.25	(5.02, 17.08)	<0.001	7.39	(3.97, 13.77)	<0.001
25-44	6.75	(4.58, 9.95)	<0.001	3.92	(2.58, 5.94)	<0.001
45-64	3.40	(2.54, 4.54)	<0.001	3.00	(2.24, 4.02)	<0.001
65+	1.00	— —	—	1.00	— —	—
Sex						
Male	1.00	— —	—	1.00	— —	—
Female	0.94	(0.78, 1.13)	0.517	0.87	(0.72, 1.05)	0.15
Ethnicity						
White British	1.00	— —	—	1.00	— —	—
White Irish	1.18	(0.49, 2.86)	0.708	0.82	(0.34, 2.02)	0.67
White Other	2.32	(1.41, 3.82)	0.001	0.71	(0.40, 1.24)	0.23
Mixed	3.23	(1.25, 8.37)	0.016	1.40	(0.52, 3.81)	0.51
South Asian	5.38	(2.19, 13.19)	0.000	1.53	(0.58, 4.0)	0.39
Other Asian	0.77	(0.15, 3.93)	0.750	0.29	(0.05, 1.50)	0.14
Black	3.35	(0.54, 20.72)	0.193	0.73	(0.10, 5.18)	0.76
Other Ethnicity	0.70	(0.08, 5.90)	0.745	0.26	(0.03, 2.32)	0.23
Household income combined for last 12 months						
£0-9,999	1.99	(0.97, 4.07)	0.060	1.40	(0.69, 2.85)	0.35
£10,000-24,999	1.00	— —	—	1.00	— —	—
£25,000-49,999	1.32	(0.90, 1.95)	0.156	1.10	(0.75, 1.61)	0.62
£50,000-£74,999	2.62	(1.68, 4.08)	0.000	1.56	(1.00, 2.44)	0.05
£75,000-£99,999	2.70	(1.54, 4.74)	0.001	1.31	(0.74, 2.33)	0.36
£100,000+	2.87	(1.63, 5.03)	0.000	1.21	(0.68, 2.18)	0.52
Prefer not to say	0.92	(0.47, 1.82)	0.812	0.64	(0.33, 1.25)	0.19
Missing	1.96	(1.11, 3.44)	0.020	1.28	(0.73, 2.26)	0.39
Geographical region						
East of England	1.00	— —	—	1.00	— —	—
East Midlands	1.28	(0.75, 2.18)	0.366	1.27	(0.74, 2.17)	0.39
London	7.80	(5.02, 12.13)	0.000	5.79	(3.60, 9.31)	0.00
North East	1.24	(0.64, 2.39)	0.525	1.30	(0.67, 2.54)	0.43
North West	1.96	(1.22, 3.15)	0.006	2.07	(1.28, 3.35)	0.00
South East	0.99	(0.65, 1.52)	0.978	0.94	(0.61, 1.44)	0.77
South West	0.56	(0.30, 1.03)	0.063	0.58	(0.31, 1.08)	0.09
Wales	0.65	(0.23, 1.82)	0.412	0.68	(0.24, 1.93)	0.47
West Midlands	1.51	(0.81, 2.84)	0.199	1.49	(0.79, 2.81)	0.22
Yorkshire and The Humber	1.76	(0.95, 3.27)	0.074	1.86	(0.98, 3.50)	0.06

Sensitivity analyses were consistent with the finding that overcrowding was associated with increased risk of PCR and anti-N total immunoglobulin assay antibody confirmed SARS-CoV-2 ( Table 5). In a sensitivity analysis where we did not subtract 1 from the number of rooms there was evidence of an increased odds of having a positive SARS-CoV-2 antigen result in balanced (OR: 2.08; 95% CI: 1.16–3.73; p-value=0.015) and overcrowded households (OR: 2.37; 95% CI: 1.16–4.83; p-value=0.017) compared with those in under-occupied houses.

**Table 5.** Sensitivity analyses of mixed effects logistic regression models.

		PCR				Antibody			
		OR	95% CI		p-value	OR	95% CI		p-value
Model A	Balanced	1.72	1.13	2.63	0.012	2.64	1.57	4.43	<0.001
	Overcrowded	2.45	1.43	4.19	0.001	3.32	1.54	7.15	<0.001
Model B	Balanced	2.39	1.69	3.38	<0.001	3.62	2.19	5.96	<0.001
	Overcrowded	3.54	2.23	5.63	<0.001	4.79	2.29	10.03	<0.001
Model C	Balanced	1.51	1.05	2.15	0.024	3.05	1.86	5.02	<0.001
	Overcrowded	2.03	1.27	3.24	0.003	4.33	2.05	9.15	<0.001
Model D	Balanced	2.13	1.30	3.47	0.002	2.46	1.38	4.38	0.002
	Overcrowded	2.40	1.22	4.75	0.012	3.40	1.43	8.06	0.006
Model E	Balanced	2.08	1.16	3.73	0.015	1.80	0.65	4.97	0.257
	Overcrowded	2.37	1.16	4.83	0.017	4.48	1.51	13.28	0.007

Model A: age, sex, ethnicity, household income and geographical region included as covariates. Model B: household income and geographical region included as covariates as a minimally sufficient adjustment sets informed by our Directed Acyclic Graph. Model C: age, sex, households with children, and number of close contacts outside the household as covariates (repeating a analysis previously reported prior to having occupational category or household income data available). Model D: sex, ethnicity, income and occupation as covariates. Model E: age, sex, ethnicity, household income and geographical region included as covariates with overcrowding exposure recalculated by only excluding one room from households with 2 or more room.

## Discussion

We estimate that overcrowded households have between 2- and 4-times the odds of PCR-confirmed SARS-CoV-2 and anti-N total immunoglobulin assay antibody positivity compared to under-occupied households. These elevated odds of confirmed SARS-CoV-2 remained after we adjusted for differences in demographic and other socio-economic factors between households that may increase risk of being infected. We also found that people in accommodation considered balanced, where the number of rooms was equal to the number of people, were at increased risk of PCR confirmed SARS-CoV-2 compared to under-occupied houses.

Virus Watch is a large national household community cohort study of the occurrence and risk factors for SARS-CoV-2 infection and is designed to estimate incidence of PCR-confirmed SARS-CoV-2 and measure effectiveness and impact of recommended COVID-19 control measures. Individuals in the study are geographically distributed across England and Wales and the cohort is diverse in terms of age, sex, ethnicity and socio-economic composition, and levels of overcrowding were comparable with national estimates for England and Wales. Respondents to our housing survey were more likely to be White British, over the age of 65 and have a higher income. As a result, our unadjusted estimates of the association between overcrowding and SARS-CoV-2 are likely to underestimate the strength of the association, as minority ethnic households have the highest rates of overcrowding. Our multivariable estimates adjust for age, ethnicity and income; however, due to the upper limit of 6 people in a household and the higher socio-economic status of participants in Virus Watch compared with the general population, our estimates are likely to underestimate the true magnitude of the association.

Several additional limitations are important when interpreting our findings. Virus Watch participants were required to reside at least four days at the same address, but as Virus Watch did not collect more detailed information on time spent with other household members inside, more granular data on intra-household contacts could be collected in the future to examine this issue further. Individuals living in nursing or residential homes were not eligible for inclusion in Virus Watch and, therefore, our results are not generalisable to these settings. The linked PCR data we included from the Second Generation Surveillance System for SARS-CoV-2 did not include lateral-flow device results and as such we will miss individuals who tested at home without a confirmatory PCR test, but note that anyone testing positive with a lateral-flow test is advised to have a confirmatory PCR test.

There were more participants taking part in the February 2021 monthly survey that were over the age of 65 compared with the general population in England and Wales, more people of White British ethnicity, and more people of household size of 2. Virus Watch is limited by the fact that only households with a lead householder able to speak English and access the internet were able to take part in the study ^15^. An important additional limitation is that only households of up to six people were eligible for inclusion. By not including households of over six people, we are likely underestimating the risk associated with overcrowding. Conversely, we cannot rule out residual confounding as a possible alternative explanation for some of the excess risk in the associations we describe.

Access to SARS-CoV-2 antigen testing is socio-economically patterned, with those in more deprived areas having less ability to access tests and less likely to be contact traced ^20^. Our data may therefore under-estimate the true infection risk associated with overcrowded housing. To mitigate this bias in access to antigen testing, we analysed antibody data from the laboratory cohort where home test kits were provided to all adult participants.

Our findings highlight the importance of public health interventions to prevent and stop the spread of SARS-CoV-2 considering the much greater risk of being infected for people living in overcrowded households. The pathways between overcrowding and increased risk of infection are not fully understood. Overcrowded households are more likely to be found in socioeconomically deprived and urban areas, and higher levels of COVID-19 in these communities include higher likelihood of working in public-facing and essential occupations ^21^, lower likelihood of working from home during lockdowns, constraints to self-isolation when ill or in contact with a case (both financial and due to lack of suitable space within an overcrowded house), increased number of household members sharing of common spaces and facilities. Overcrowded households are also more likely to suffer from environmental exposures that have been linked to COVID-19 including air pollution ^12^.

Overcrowding is an important risk through which COVID-19 inequalities manifest. To address social, ethnic and regional inequalities in COVID-19 outcomes, public health responses should explicitly address overcrowded housing. Infectious cases in overcrowded houses are less likely to be able to be isolated from other household members or to avoid using shared spaces, including bathrooms. However, existing advice on the importance of infectious cases using masks and ventilation through opening of windows should be supported by tailored communications strategies. Schemes that provide hotel accommodation for cases with vulnerable residents in overcrowded households, such as those used internationally ^22, 23^ and recently introduced in the London Borough of Newham, should be considered a priority public-health intervention. Such community quarantine and isolation options require clear, inclusive guidance with specific advice for large households and multigenerational families. Although the UK does not target COVID-19 vaccine according to social factors, our results highlight the need to ensure high vaccine coverage in areas with high levels of overcrowding. There is currently evidence of lower COVID-19 vaccination rates in such areas, highlighting the importance of working closely with local community groups to increase vaccination intention and ensuring vaccination is highly accessible.

Improving the standard and supply of housing in the UK is important for the COVID-19 recovery as 32% of all households are estimated to experience overcrowding, affordability challenges or non-decent housing that influence their risk. In relation to these challenges, a paper by the ethnicity sub-group of the Scientific Advisory Group for Emergencies (SAGE) recommends the following measures to reduce the risk of transmission within households: provision of emergency grants for repair and maintenance of social and private rental housing, particularly to increase ventilation; removing the benefit cap and welfare restrictions, particularly in high housing cost areas and for immigrant families; reviewing implementation of the bedroom tax for those in multi-person households; and investment in affordable childcare and alternative community spaces for social connection, particularly for the elderly.

Addressing England’s overcrowding challenge is complex and requires action from multiple government and private sector stakeholders, including the following: revising the statutory overcrowding standard, increasing the housing supply and size of homes/rooms, and reducing under-occupation in social housing ^24^. Breaching the statutory overcrowding standard is a criminal offence, yet the threshold is considered to be too low such that very few homes are statutorily overcrowded, reducing local authorities’ ability to take action ^24^. Housing supply has fallen short of demand for decades ^25^, which is caused by inter-related factors, such as construction capacity, planning regulation, public opposition to new development, overseas buyers, overreliance on the private sector and underuse of social approaches to housing development ^26^. English homes are among the smallest in Europe ^27^ and the Nationally Described Space Standard ^28^ for new homes is only mandatory if implemented through local planning policies following tests for need and viability. Finally, although there are under-occupied and empty homes in England, these are not suitable to solve the housing supply problem due to their location, ownership and other factors.

Housing is an important determinant of health for many physical and mental health conditions and COVID-19 has exposed, in real-time, vulnerabilities in the English housing stock, particularly for low-income and minority ethnic populations. Looking forward, the country’s health and resilience to future pandemics and other risks, such as climate change ^29, 30^, are dependent on the state of the housing stock. This analysis of overcrowding and COVID-19 underscores the need to improve housing for health. As part of the agenda to “Build Back Better” ^31^ and fairer ^32– 34^, investment in sustainable, high-quality and more affordable housing will support health, jobs and the wider economic recovery so that we are prepared for the key challenges of the 21st century.

## Data availability

### Underlying data

We aim to share aggregate data from this project on our website and via a "Findings so far" section on our website - https://ucl-virus-watch.net/. We are sharing individual record level data on the Office of National Statistics Secure Research Service, and given the sensitive content in our dataset (information on income and household characteristic) for this study, we currently cannot release the data at the individual level. In sharing the data we will work within the principles set out in the UKRI Guidance on best practice in the management of research data. Access to use of the data whilst research is being conducted will be managed by the Chief Investigators (ACH and RWA) in accordance with the principles set out in the UKRI guidance on best practice in the management of research data. Data access requests to data can also be made directly to the Virus Watch chief investigators (ACH or RWA) at the following email address: viruswatch@ucl.ac.uk.

### Extended data

Zenodo: Extended data for ‘Household overcrowding and risk of SARS-CoV-2: analysis of the Virus Watch prospective community cohort study in England and Wales’ https://doi.org/10.5281/zenodo.5708766
