# Supplementary material for: Household overcrowding and risk of SARS-CoV-2: analysis of the Virus Watch prospective community cohort study in England and Wales
Source: Wellcome Open Res. 2021 Dec 15;6:347. [Version 1] doi: 10.12688/wellcomeopenres.17308.1 (PMC11130583; doi:10.12688/wellcomeopenres.17308.1)
Supplement: Peer review history

# Reviewer report: Reviewer response for version 1

**DOI:** 10.21956/wellcomeopenres.19135.r73487

**Published:** 2024-05-27

**Authors:** Eben Kenah

**Affiliations:** 1 Division of Biostatistics (College of Public Health), The Ohio State University, Columbus, Ohio, USA

It is great to see the implementation of a new generation of Virus Watch studies. Although I have several comments about the statistical analysis, the overall conclusions of the study appear sound. Its most important limitations are discussed adequately, and its implications for public health are clearly explained.

 - Overcrowding measure: The categorization of the number of people per room is not sufficiently justified. Why not use the original variable and fit a flexible model using orthogonal polynomials or splines? Odds ratios could be reported comparing specific levels of the people/room measure, which might be easier to interpret than the comparisons of overcrowded and balanced households with und-occupied housing. The under-occupied and overcrowded categories are mixtures of households at different levels of crowding.

 - Covariates: The number of individuals in the household is likely to act as a confounder of the association between overcrowding and the risk of SARS-CoV-2 infection. Even if we imagine that the transmission probability between household members is constant in any given crowding category, a larger household can support longer chains of transmission and a higher risk of infection from within-household transmission. Although some of this confounding may be attenuated by the household-level random effect, it would be better to include household size as a covariate. The resulting model would isolate crowding as an exposure more effectively. However, I doubt that this would affect the qualitative conclusion that crowding is associated with higher risk of infection.

 - Results: Given that the vast majority of the study population lives in "under-occupied" housing, it seems like the categories used in the study might not be a great fit for the context. Besides using the original people/rooms variable (see above), another alternative would be to base the categories on quantiles of the crowding measure. I acknowledge that the categories reported here match those used in earlier research on overcrowding, but I am not sure they are the best choice for the question being addressed here.

 - Figure S1: The explicit use of a DAG to identify possible confounders is commendable, but the DAG would be much easier to read and interpret if a consistent direction of time was maintained (e.g., left to right or top to bottom). The DAG has arrows pointing left, right, up, and down.

 - R code: The code is well-formatted and readable. My only comment is that profile or bootstrap confidence intervals are generally better choices than Wald confidence intervals. Is there a reason that these were not used?

Is the work clearly and accurately presented and does it cite the current literature?

Yes

If applicable, is the statistical analysis and its interpretation appropriate?

Partly

Are all the source data underlying the results available to ensure full reproducibility?

Yes

Is the study design appropriate and is the work technically sound?

Yes

Are the conclusions drawn adequately supported by the results?

Yes

Are sufficient details of methods and analysis provided to allow replication by others?

Yes

Reviewer Expertise:

epidemiologic methods, survival analysis, causal inference, statistical methods for infectious disease epidemiology, epidemic models, networks

I confirm that I have read this submission and believe that I have an appropriate level of expertise to confirm that it is of an acceptable scientific standard.

# Reviewer report: Reviewer response for version 1

**DOI:** 10.21956/wellcomeopenres.19135.r71408

**Published:** 2024-05-25

**Authors:** Kathy Leung

**Affiliations:** 1 The University of Hong Kong, Hong Kong, Hong Kong

The authors presented an analysis of SARS-CoV-2 infection risk using data from the Virus Watch study in the UK. The design of the Virus Watch study is robust, and it is an advantage that the survey results can be linked to the SARS-CoV-2 testing results in the national database. However, I have reservations about the design and selection of regression model of this study. Please see below for my major comments:

1. The definition of overcrowding relied only on the calculation of the number of household occupants divided by the number of rooms but did not consider the composition of household occupants. For example, households with school-aged children tend to be more crowded, and school-aged children are considered to be the driver of transmission in many respiratory infectious diseases. It is therefore difficult to separate the effects of overcrowding and having one or more school-aged children in the households.
2. Similarly, the study was carried out between June 2020 and February 2021 and the UK experienced several extended periods of lock downs. I presume lock down would increase the time residents spent at home but there should be a differential effect for people with different working statuses. The occupations of household members and whether they were able to work from home during lock downs should be considered in the model.
3. Since the mass vaccination programme started in late 2020 and/or early 2021, the model should also consider the differential vaccination coverage across different age groups. The older adults aged 65 or above were prioritised in early 2021 in the vaccination programme.

Is the work clearly and accurately presented and does it cite the current literature?

Yes

If applicable, is the statistical analysis and its interpretation appropriate?

Partly

Are all the source data underlying the results available to ensure full reproducibility?

Yes

Is the study design appropriate and is the work technically sound?

Yes

Are the conclusions drawn adequately supported by the results?

Partly

Are sufficient details of methods and analysis provided to allow replication by others?

Yes

Reviewer Expertise:

infectious disease modelling

I confirm that I have read this submission and believe that I have an appropriate level of expertise to confirm that it is of an acceptable scientific standard, however I have significant reservations, as outlined above.

# Reviewer report: Reviewer response for version 1

**DOI:** 10.21956/wellcomeopenres.19135.r51869

**Published:** 2022-09-01

**Authors:** Melissa Rolfes

**Affiliations:** 1 Influenza Division, Centers for Disease Control and Prevention, Atlanta, GA, USA

**Summary:**

 The authors describe an analysis of SARS-CoV-2 infection risk among a community cohort in the UK. This is a long-standing cohort with study procedures and activities that pre-date the COVID-19 pandemic. Participants who completed a monthly survey in February 2021 were considered for analysis. These participants provided demographic and household characteristics as well as vaccination information. Household information was used to classify participants as living in under-occupied, balanced, or overcrowded settings based on the number of people per rooms in the household. The risk of SARS-CoV-2 infection was compared between these different living situations using two outcomes: 1) evidence of acute SARS-CoV-2 infection using information from linked national COVID-19 testing information systems and 2) evidence of acute infection from anti-N antibodies collected from a sub-set of participants. Using these two outcomes the authors found that participants living in over-crowded settings (with greater numbers of people per room) and those living in balanced settings (with equal numbers of people per room) had significantly greater odds of SARS-CoV-2 infection. This evidence stood up to several sensitivity analyses which examined the impact of assumptions and potential confounders.

 The authors conclude that there should be greater investment in reducing the occurrence of overcrowding and also in providing means to prevent and control transmission of infectious diseases within overcrowded settings. This type of investment may need to be large but also innovative as individuals living in overcrowded settings are at additional social and economic disadvantages that impact uptake and success of common public health measures.

**Major comments:**

- Background would benefit from a statement or two about what this study provides that prior literature doesn’t. The authors point to many studies (retrospective and prospective) to demonstrate greater incidence of COVID-19 in households with a greater density of people. I think it would be helpful to highlight what gaps this study and analysis fill.
- In the first paragraph of the Methods section, it would be helpful to state the study procedures at a high-level. There are very detailed study procedures in other publications, but a few broad points would be helpful here, too. I was looking for information about inclusion/exclusion criteria, duration of recruitment (recruitment began in June 2020, when did it end or is it rolling?), anticipated duration of follow-up when participants enroll, etc. The main webpage for the Virus Watch study has a nice, concise summary that has these details. I strongly encourage the authors to modify this Methods paragraph to include additional information.
  - The Discussion section does include some inclusion/exclusion criteria, but I strongly encourage the authors to move those details to the Methods.
- Additionally, in the methods, it would be helpful to know if participants needed to enroll along with other household members or if participants could enroll alone (even if their other household members did not enroll). I think adding in the inclusion/exclusion criteria would be helpful to address this confusion.
  - This information also has relevance for the interpretation of the household characteristics in Table 1 and Table 2. If multiple people per household were allowed to participate, then there is a correlation induced in the presented proportions and would not necessarily be able to be compared with the ONS summary data.
- Some readers may not be familiar with the UK test and trace program. Please include in the Methods (rather than in the Discussion) a few details about the type of laboratory assay that was be conducted. was it a molecular/PCR assay or a rapid antigen assay? If it was a rapid antigen test, were positives confirmed by PCR? There is some use of “PCR” and some use of “antigen test” in the Results section that could create confusion without some further details.
- It is not clear whether households with just 1 member were included in analysis. It seems like including participants who do not have another household member sharing their living space would have different risk from different places than those who did share their living space. Suggest including information in the Methods about how single-person households were managed in the analysis. And if they were original included, I suggest the authors include a sensitivity analysis excluding the single-person households.
- The authors describe a sub-cohort of ~10,000 participants who provided capillary blood specimens for antibody testing. It would be helpful for readers to see a column in Table 1 describing these 10,000 participants in the sub-cohort. This level of detail would help the reader assess confounding, as the magnitude of the association between SARS-CoV-2 and overcrowding was greater when the outcome was antibody results. As a reader, I wonder whether the demographic and household characteristics differed in the sub-cohort and could account for some of the differences in the magnitude of the association with overcrowding.
- In Table 2, the results of the PCR tests are reported, by household and demographic characteristics. The PCR-negative and not tested group are combined into one, however, this “lumping” is potentially misleading. There are often social and demographic features associated with getting tested for SARS-CoV-2 and those associations would be masked if these two groups are lumped together. I suggest the authors consider making 3 columns for the PCR test results in Table 2: Not tested | Negative | Positive. If it is not possible to separate these two groups (negative and not tested) because of the way that the test and trace program reporting was done, please add that detail to the Methods to inform readers of the reason behind lumping.
  - Furthermore, for the regression analysis, the decision could be made to lump negative with not tested, but the reader isn’t able to assess the viability of that lumping without seeing the two groups split in Table 1.  
- The authors describe that information on COVID-19 vaccination was collected from participants but have not included vaccination in the analysis. Given that vaccines during this time period (Feb–Aug 2021) were well-matched to the circulating SARS-CoV-2 variant and provided meaningful protection against infection and that vaccination uptake could be associated with overcrowding (as the authors point out in the Discuss), I strongly encourage the authors to include vaccination as a covariate in the regression models.

**Minor comments**

- In Table 1, ONS is used as an acronym but doesn’t seem to have been previously defined.
- In Tables 3 and 4, the p-values are presented with inconsistent significant digits but would be helpful to be consistent.
- In the Discussion, there are several links to websites with information on public health control measures and further guidance. These should be included as numbered references rather than as hyperlinks within the text. This change would enable those readers with only a text pdf or limited internet access to reach these linked urls.

Is the work clearly and accurately presented and does it cite the current literature?

Yes

If applicable, is the statistical analysis and its interpretation appropriate?

Yes

Are all the source data underlying the results available to ensure full reproducibility?

Yes

Is the study design appropriate and is the work technically sound?

Partly

Are the conclusions drawn adequately supported by the results?

Yes

Are sufficient details of methods and analysis provided to allow replication by others?

Partly

Reviewer Expertise:

Epidemiology, influenza, transmission

I confirm that I have read this submission and believe that I have an appropriate level of expertise to confirm that it is of an acceptable scientific standard, however I have significant reservations, as outlined above.
